# What leadership role should University Medical Centers take in regional primary prevention networks? An interdisciplinary, multi-method analysis of a crowded stakeholder environment

**DOI:** 10.1371/journal.pone.0305262

**Published:** 2024-07-11

**Authors:** Marlot Kuiper, Scott Douglas, Julie Keunen, Helene Voogdt-Pruis, Lilian van der Ven, Diederick Grobbee, Yvonne van der Schouw

**Affiliations:** 1 Faculty of Law, Economics and Governance, School of Governance, Utrecht University, Utrecht, The Netherlands; 2 Julius Center for Health Sciences and Primary Care, University Medical Center Utrecht, Utrecht University, Utrecht, The Netherlands; Kilimanjaro Clinical Research Institute, UNITED REPUBLIC OF TANZANIA

## Abstract

Advancing public health through prevention necessitates collaboration among public, private, and community actors. Only together can these different actors amass the resources, knowledge, and community outreach required to promote health. Recent studies have suggested that university medical centres (UMCs) can play a key role in regional prevention networks, given their capacity to initiate, coordinate, drive, and monitor large partnerships. Yet, the literature often refers to prevention activities in general, leaving underexplored what UMCs can add to *primary*, *universal* prevention networks specifically. Moreover, UMCs operate in a crowded field of other organizations with extensive experience in primary prevention, who will already have an idea about what role UMCs should play in the network. This article presents a case study examining the potential role of a UMC within a densely interconnected stakeholder environment in the surroundings of a large city in the Netherlands. Combining insights from public health studies and network governance research, and integrating data from various methods, this study concludes that UMCs can enhance their contributions to prevention by assuming the role of network servants rather than network leaders. Stakeholders consider public health authorities or municipal governments as more logical candidates for coordinating the network. Moreover, partners often perceive–deservedly or not–UMCs as overly focused on the medical aspects of prevention, potentially neglecting social interventions, and as favouring universal treatments over tailor-made community interventions. At the same time, partner organizations hope that the UMCs join collaborations within the community, using their expertise to measure the impact of interventions and leveraging their prestige to generate attention for primary prevention. By synthesizing theoretical insights from multiple disciplines and analysing the empirics of network leaderships through multiple methods, this study offers UMCs a contextually-informed perspective on how to position themselves effectively within primary prevention networks.

## Introduction

Patients in OECD countries increasingly present with non-communicable chronic diseases like cancer, cardiovascular diseases, and depression associated with health risk behaviours such as lack of physical activity, unhealthy diet, and alcohol and drug use [1–3]. It is therefore acknowledged that preventing illness and supporting healthy lifestyles ‐ rather than ‘treating disease’ ‐ is essential for pushing back diseases and controlling healthcare costs [4]. The Covid-19 pandemic further underlined the importance of universal, primary prevention, as a large share of the patients that ended up in the intensive care unit in OECD countries were over-weight [5].

This shift towards primary universal prevention necessitates collaborative efforts in regional networks beyond traditional partnerships [6]. No actor can individually possess all the knowledge required, affect all the lifestyle interventions desired, and reach all parts of the community required for an effective regional prevention program. The healthcare sector has traditionally been dominated by organizations such as hospitals, primary care providers, and pharmaceutical companies. As healthcare expands to include a more pro-active, preventive approach, the collaboration with actors such as local governments, supermarkets, schools, and community organizations becomes important [4, 7, 8].

Regional collaboration is crucial, but networks are often deemed “too fragmented and depending on the commitment and capacities of individuals” [5] to be effective. Recent research publications and policy papers have argued that university medical centres (UMCs) could play key roles by coordinating the various actors involved [9–11]. University organizations are generally seen as suitable ‘anchor institutions’ for community advancement, due to their established position among local stakeholders [10, 12, 13]. UMCs specifically are described as “classic professional institutions” [14–16] capable of disseminating available knowledge to local initiatives [11]. Hence, UMCs are increasingly given key roles as ‘leaders’, ‘network coordinators’ or ‘quartermasters’ to initiate, coordinate, drive, and monitor prevention networks [11].

Traditionally, UMCs have focused on secondary and tertiary prevention activities, enjoying a natural leadership role in those areas. However, it remains unclear what roles UMCs should play within primary universal prevention networks targeting the broader population [17]. In engaging in primary prevention networks, UMCs have to navigate a crowded field of established organizations–such as the local government, public health authorities, and community groups ‐ who have been working on primary prevention for years. Any move by one of the stakeholders is an intervention within a tangled web of institutions [18]. And over the years, these actors will have formed set opinions about the other organizations, including the local UMC. For example, actors like general practitioners, schools, and social workers might associate the UMCs more with ‘treating’ rather than with ‘preventing’ diseases, and more with generating generalizable knowledge than with unearthing locally relevant, pragmatic insights.

The context and history of the prevention network constrain how the regional collaboration can be organized and how it can be led [19]. In this study we use insights from public health studies and network governance research, and collected data through multiple methods, to better understand the perceptions among partner organizations of the role of UMCs in primary prevention networks. The central research question is: *What leadership roles are partner organizations willing to grant to UMCs in primary prevention networks*? Gaining a more nuanced understanding of these expectations will assist UMCs in positioning themselves within regional primary prevention networks, either as network leaders driving initiatives or as facilitative network partners fostering collaborations.

## Methods

### Case study: Primary prevention networks in a Dutch region

In this paper we present a case study of regional prevention networks surrounding a large city in the Netherlands. We hence studied regional collaboration on prevention, with a specific focus on the role and activities of the UMC within those networks. We see networks as fundamentally important empirical units for understanding the organization of preventive solutions. Throughout the research process we identified what partnerships were active in the region and bounded our case accordingly [20]. In line with the aforementioned importance of context and history of networks, this study provides a thick-description of this specific case. We do not strive for empirical generalizations, but do expect to identify mechanisms which could apply in other contexts as well [21].

#### The Dutch context

The Dutch healthcare system is characterized by universal coverage through mandatory insurance. All Dutch residents are required to purchase basic health insurance from private health insurers. Insurers are legally required to accept all applicants. The system operates under a regulated competition model, with a mix of public and private providers. Patients, health insurers and providers determine price, quality and service based on supply and demand, within regulations set by the government [22, 23].

The Dutch healthcare system is mainly governed by five key laws. The Public Health Act regulates the organization of public healthcare, including infectious disease control and population screening. The Social Support Act and Youth Act oblige local governments to provide support and assistance to (potentially) vulnerable people and youth. Lastly, the Health insurance Act regulates basic health insurance coverage and access to essential medical care while the Long-Term Care Act regulates long-term care for people with significant care needs [23, 24]. With regard to prevention, primary prevention generally falls within the framework of the first three laws, while secondary and tertiary preventive interventions are in the domain of the last two laws [25].

Financing of the healthcare system is primarily public through income taxes, government grants, and insurance premiums [23]. Total healthcare expenditure for 2021 is estimated at 96.6 billion euros, or 11.1% of GDP [26]. An estimated 2.6% of total expenditure is spent on preventive care [23].

#### The role of UMCs in prevention

In the Netherlands, UMCs have been formally awarded a lead role in regional prevention networks. In 2019, the Dutch Federation of University Medical Centres, commissioned by the minister of medical care, laid down an ambitious plan for regional prevention networks. Each of the seven UMCs would initiate, facilitate, coordinate, and monitor a network involving elderly care, general practitioners, municipalities, public health services, and insurers to advance prevention in their home region [11]. This emphasis on prevention networks is part of a wider focus on collaboration in the Netherlands, leading to active but also crowded stakeholder fields [27].

Within our case we focus on a specific, a part the general university with a long history of medical education since the 1600s. Today, the UMC boasts more than 12,000 employees, educates over 4,000 students, and serves over 230,000 patients annually. Situated at the heart of the region with over one million inhabitants, it operates amidst a multitude of public, private, and community organizations dedicated to public health. These entities range from long-standing religious care organizations to tech-driven startups promoting healthy behaviours. Local and regional governments in the area actively promote numerous networks and platforms, creating a complex environment for the UMC to navigate [28].

### Data collection

We used a three-staged iterative design to collect data among the partner organizations to analyse their view on the role and leadership activities of the UMC in the primary prevention network.

#### Phase 1: Survey

In phase one we conducted an online survey, using Qualtrics XM software, to ask the network actors about their prevention activities, their interactions with the various partner organizations, and their views on the distribution of the leadership roles.

In preparation for the survey, we mined the grey literature to draft a set of different primary prevention initiatives stakeholders could be engaged in, ranging from supporting lifestyle interventions, educating professionals that are associated with primary prevention, or developing technological solutions. We then asked what activities the respondents were involved in. Finally, we derived four types of *leadership roles* from the network literature to query partners about which actors within the collaboration take these roles, and which roles the UMC should be taking in particular. The four activities were:

- **Initiating collaborative action.** Convening the various partners around a common purpose. Scholars link this type of activities to roles like network *convenor* [29] or *anchor* [12, 13, 30].
- **Coordinating collaborative action.** Coordinating the work of the various partners by keeping track of who is doing what. Scholars link this type of activity to roles like *network manager and*, *steward* [31, 32].
- **Driving joint action and solutions.** Making sure the network delivers by creating momentum for action and driving creative solutions. Scholars link this type of activity to a role like *network catalyst* [31–33].
- **Monitoring** of results. Collecting information about the outputs and achievements of the network and assessing whether these are satisfactory. Scholars links this type of activity to a role of *network evaluator and monitor* [28].

We tested the scope and clarity of the survey questions with the panel of local public health professionals supporting the study, rephrasing questions and adding answers options where needed. We used the hybrid name generator method [34] to support the validity of our measure. We provided respondents with a free-call measurement instrument (Q5: “Name a maximum of three organizations in the Utrecht region with which your organization collaborates most frequently and intensively on prevention. Describe the activities you undertake together.”). Respondents were also repeatedly prompted with a roster to indicate their collaboration with predefined organizations (Q7: How often do you interact with the organizations below regarding universal primary prevention?– 12 types of organizations listed, including ‘other’). This method helps to overcome the recall problems associated with the free-recall name generator, but also reduces the bias toward only naming actors included on a roster [34].

In correspondence with the public health professionals involved, we targeted a cross-section of 183 different types of public, private, and community actors active in the network. We distributed the survey through contact information the UMC had available and through the municipality and public health service. Data was collected in line with the GDPR and all survey participants explicitly provided their consent at the start of the online surveyFinally, we explicitly invited partners to further distribute the survey to actors who they deemed relevant for this study. We sent out two reminders, and contacted target respondents by phone. Ultimately, 75 respondents participated in the survey (40% of the targeted organizations), of whom 45 fully completed the survey (full response rate = 60%). As many respondents did complete the first part of the survey on prevention activities and collaborations but did not finish the survey, the analysis in this paper is based on both the incomplete and fully completed surveys.

#### Phase 2: Semi-structured interviews

We used purposive sampling [35, 36] to select a variety of respondents from the survey to approach them for in-depth interviews. We selected respondents from a variety of organizations, and in different functions (e.g. social worker, researcher, insurer, general practitioner, municipality, public health service). We reached out to 14 respondents with an email explaining the scope of our research project and asking them to participate, with all 14 respondents consenting to an interview. The interviews, lasting 45 to 70 minutes, were conducted in person or via MS Teams. The interviews were semi-structured, which provided a framework but also gave us the flexibility to probe on certain issues with more open and follow-up questions [37]. The interviews were conducted by the first, second, and third authors in pairs. After each interview, the second interviewer drafted the report, which the first interviewer then reviewed, and finally all respondents were asked to approve the report.

#### Phase 3: Online working conference

As a final step, we organized a one-hour online working conference to verify and sharpen or preliminary findings. We invited all stakeholders by email who had been invited to participate in the survey in phase two, again explaining how the data collected at the meeting would be used. We optedfor an online conference to make it accessible for busy partner organizations across the region. 35 external stakeholders participated in the conference. The research team first presented the preliminary findings, whereafter those findings were discussed in three smaller subgroups.

### Data analysis

A hybrid method of inductive and deductive thematic analysis was used to analyse the data [38]. Three research team members (1^st^, 2^nd^, 3^rd^ authors) created an initial coding scheme. This deductive approach produced a set of a priori codes that came from the theory, our research questions and the initial survey results. For instance, the network activities that were deduced from the network governance literature ‐ initiating collaborative action; coordinating collaborative action; driving joint action and solutions; and monitoring of results–were used as initial codes. Informed by both our research question and the survey results ‘collaboration with the UMC’ was a code we deduced, since we learned that stakeholders had little clue with which department of the UMC they were collaborating.

In the next stage, the first author carefully reviewed the interview reports and independently coded the data, after which coding was discussed and cross-checked with the other researchers throughout several meetings. The team for instance discussed the code ‘pragmatic knowledge’ as this links to tailormade solutions, but we felt that the data pointed more towards the kind of knowledge that actors deemed relevant for preventive solutions. This inductive work resulted in a series of post-empirical codes derived from the fieldwork, generating new themes emerging from the data. We used Nvivo14 software to code the interview reports.

To minimise the risk of bias and promote study rigour, the following steps were followed: we documented an a priori methodology; two or more members of the research team conducted the interviews and data analysis; Findings were discussed and documented at each stage via digital and face-to-face meetings.

### Ethics

As this study did not involve patients or study participants, an ethical research approval was not needed according to Article 1b of the Dutch Medical Research in Human Subjects Act. However, the Dutch Code for Scientific Integrity did still apply, meaning we sought informed and explicit consent from all respondents at the various stages of the research (as detailed above), analysed the data in line with the Code and GDPR guidelines. All respondents could also withdraw from the study at any time.

## Results

### A crowded stakeholder environment

The survey and subsequent interviews showed that primary prevention is a crowded field. A variety of partner organizations participated in this study. Table 1 provides an overview of the stakeholders that participated in this study.

**Table 1:** Overview of research participants.

Organizations (alphabetical order)	Phase 1: Survey *February and March 2022*	Phase 2: Semi-structured interviews *May 2022*	Phase 3: Online working conference *June 2022*
First-line care organizations	7		1
Government (e.g. municipalities)	14	2	4
Health insurances	3	4	3
Independent consultancies	2		1
Interest groups	10		
Nursing homes	4		2
Public Health Services	3	2	2
Research and/or educational institutes	15	4	7
Second- and third-line care organizations	7		7
Sport associations	4	1	1
Welfare organizations	6	1	4
Others			3
**N =**	**75**	**14**	**35**

**Fig 1:**
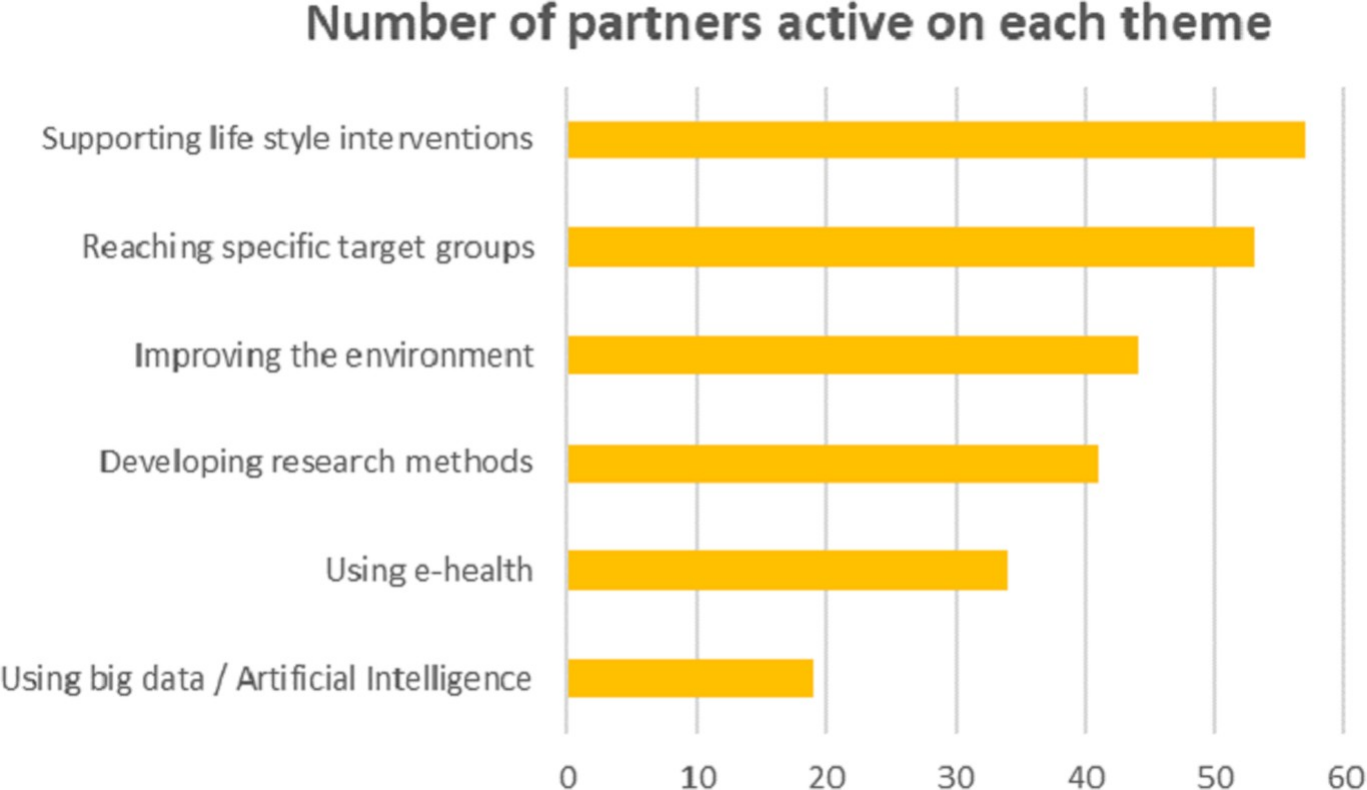
Number of participants active on each prevention theme.

Most of the respondents indicate that they perform activities that are directly aimed at prevention and supporting health; 72% (N = 75) indicates that their organization supports lifestyle interventions, 67% targets specific groups (i.e. youth, groups with a lower socioeconomic status), and 56% works on a healthy living environment. Activities that indirectly target prevention, like educating professionals, gathering data and developing research methods, are performed less often. For instance, only 23% of the organizations uses Artificial Intelligence (AI) in the delivery of preventive services (Fig 1).

Respondents collaborate on prevention with a variety of stakeholders. The municipality is the most frequent collaboration partner; 56% of the respondents that work on lifestyle interventions collaborates with the municipality. The public health service and primary care institutions are also frequent collaboration partners. Respondents also report that they are in involved in a wide range of formal and informal networks in the region.

### Interactions with the UMC

#### Individual collaborations versus the big institution

Survey respondents indicated they work with specific departments or research centres within the UMC (N = 17), such as cardiology or the cancer care. However, many respondents were unsure about the exact UMC department they collaborated with. Interviews revealed collaborations with individual professionals on small-scale projects initiated by enthusiastic experts from various institutes who know each other well, emphasizing the ease of working with *“familiar faces”*.

Despite enthusiasm about these individual professionals, respondents expressed less positive sentiments about the UMC as a whole. A respondent said: *“Sometimes you have these brilliant collaborations with individual doctors or teams*, *whilst there seems to be no organizational support from the UMC at all*.*”* The UMC’s reputation was seen as primarily cantered on tertiary care, despite its potential to generate attention for primary prevention: *“As an institution*, *the UMC does not express itself sufficiently in favour of primary prevention*. *They see themselves too much as a care institution”*.

#### Treating patients versus promoting health

A majority of the respondents argue that the UMC still has a rather narrow view on ‘preventing if not simply treating diseases’ while they themselves emphasize a more holistic perspective on ‘promoting health’. Respondents observe, for example, that ‘treatment-oriented’ professionals are used to letting people come to them, whilst health-oriented social workers proactively venture out into communities. Stakeholders expect it will take effort for the UMC to become a constructive network partner, as its professionals would need to develop different and broader conceptions of ‘prevention’ and ‘health’. A stakeholder explained: *“A transition from ‘care’ to ‘health’* …, *that’s quite a change for many health care professionals*!*”* At the same time, actors stress that an active involvement of the UMC is crucial, either out of pragmatic concern, as the UMC is a powerful partner in fund-raising, or for more substantive reasons, as ultimately actors strive towards a common goal of healthy societies.

As it stands, stakeholders argue that the UMC, despite positive exceptions, still has a predominantly *medical* perspective. Therefore, the UMC is not automatically trusted to drive a primary prevention agenda focused on ‘supporting public health’. Respondents point at the medial discourse that the UMC powerfully propagates and argues this overshadows investment in schools and social work that could also promote health. As a respondent commented, *"There is dominance from medical care*. *The social domain feels misunderstood and degraded”*.

#### Scientific knowledge versus pragmatic knowledge

A supposed strength of UMCs is command of academic knowledge, but the research shows how actors do not always value this type of knowledge. Respondents observe that for the medical researchers working in the UMC, ‘valuable knowledge’ is ‘universally true knowledge’; scientific insights gained through for example clinical trials. However, to those partners working in neighbourhoods on specific challenges in the region, valuable knowledge is context-dependent and actionable know-how. A respondent argued: *“We should look way more at what starts in neighbourhoods*, *and look at ‘what works’ from a residents’ perspective*.*”* An applied researcher claimed that the UMC *“should be open to different kinds of knowledge”*.

At the same time, partners also look to the UMC for authoritative knowledge, seeking help from the UMC when it wants to establish whether all the different prevention efforts have a societal impact. Especially, the municipality and public health service are eager to know what works. This need for evidence drives partners to the UMC as a research institution, but at the same time this type of academic knowledge is considered flawed and impractical.

#### The roles granted to the UMC

**Fig 2:**
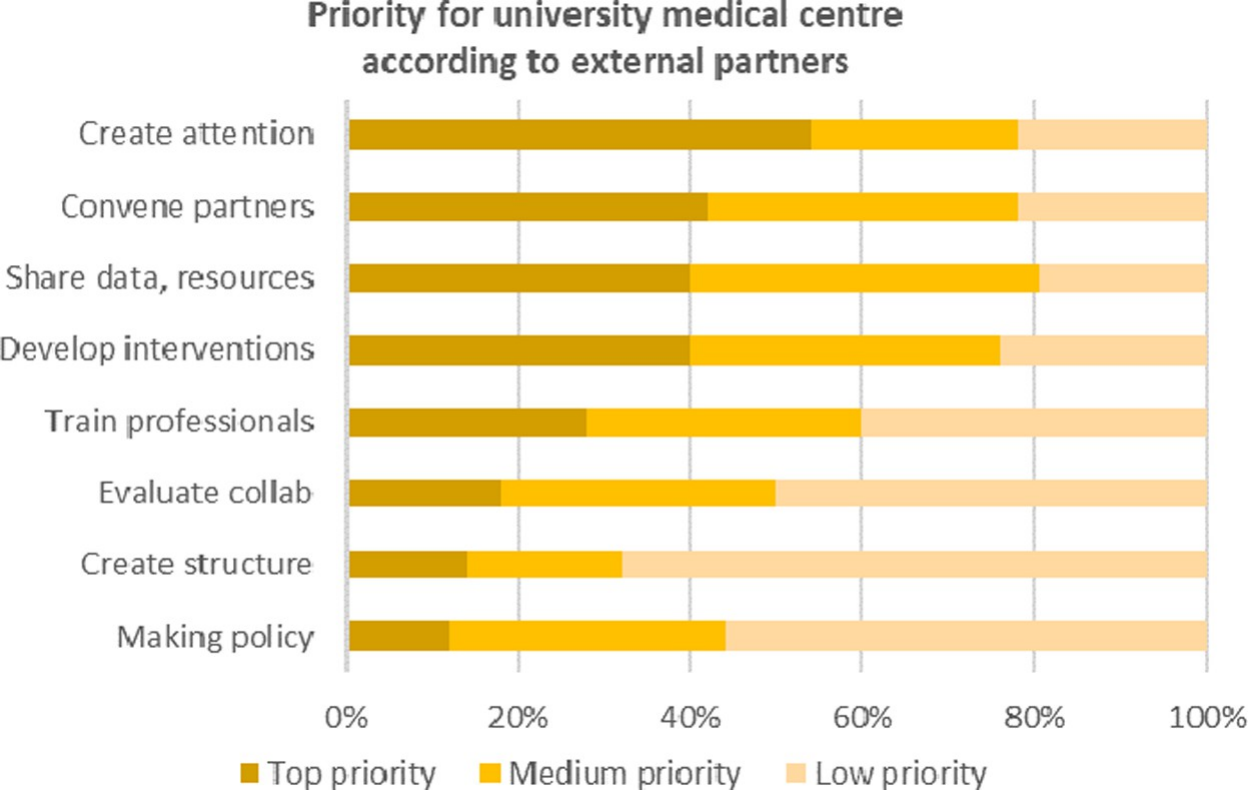
Priorities for UMC according to partner organizations.

Finally, in the survey and interviews, we asked respondents what activities and roles the UMC should prioritize within their work for regional prevention collaborations (see Fig 2). In terms of network activities, the respondents overwhelmingly did *not* want the UMC to take up leadership activities like initiating, coordinating, or driving the primary prevention network. Instead, the respondents felt that the local government or regional public health service would be a much more suitable partner. The respondents did feel that the UMC could help the network by developing smart tools to measure the impact of the collaboration on health in the region.

More substantively, partners wanted the UMC to support the network by sharing knowledge and information, and by developing innovative prevention interventions. The key priority was developing and promoting more lifestyle interventions, with respondents also see an important role for the UMC in promoting E-Health, educating professionals, and developing big data and AI tools. Fig 2 shows the priorities for the UMC according to the external stakeholders.

At the online working conference at the end of the data collection, we presented the preliminary results to a mixed group of stakeholders. The five UMC professionals participating on one hand understood the preference of the local field to nominate other organizations to lead the field. One the other hand, they did express frustration with the enduring image of UMCs as ‘treatment hospitals’. The UMCU professionals present felt a disconnect between their efforts in re-orienting the UMC internally and how the institution is perceived externally. The UMC is perceived as ‘a big hospital’, while they try to push this image towards ‘school of health’. Medical doctors are depicted as ‘aloof’, while they strive to be a supportive partner to the external environment. The UMC is described as ‘medical science focused’ while they are interested in interdisciplinary problem solving.

Towards the end of the session, both representatives from the UMC and stakeholders, cited the multiple collaborations that did indeed put these principles into practice and truly intertwined the work of the UMC and the partners in the community. Respondents observed that the image of the UMC may be the product of the past and that bad experiences with individual doctors or departments undermine the repositioning of the UMC as a whole.

## Discussion and conclusion

### Key findings and confrontation with the literature

This study explores what roles partner organizations are willing to grant UMCs in primary prevention networks. The regional partners clearly state they see only a supportive role for the UMC for three of the four leadership activities. *Initiating* partnerships in primary prevention is discouraged primarily due to the abundance of existing connections. Similarly, partners believe that UMCs are not suitable for *coordinating* these networks, as more logical options exist (the municipalities or the public health authority). UMCs are also discouraged from *driving* the collaboration because their academic focus may not align with the practical needs of local partners. And there is a concern that UMCs may prioritize "treatment" over "prevention" partners. Partners do see the UMC’s play a leading role in *monitoring* the impact of prevention networks, leveraging their research expertise.

At the same time, partners are very clear that they feel the UMC is a crucial partner within primary prevention networks, as they demonstrate by involving the UMC in their networks. Similarly, they think the UMC is crucial for well-coordinated networks that can deliver results, they just note that the UMC that the UMC should not dominate these activities. Positive examples show that egalitarian, broadly-focused, and pragmatic collaborations with regional partners are possible.

When confronting this studies’ findings with the literature, our study makes three important contributions to the literature. First, we see that whereas literature champions university hospitals as the logical choice ‘anchor institutions’ playing a lead role in networks [39–41], this study on primary prevention supporting public health paints a nuanced picture which emphasizes the role of network context and history. This study in the context of the Dutch healthcare system suggests that even though partner organizations recognize the capacities of UMCs, there not always is a *need* for their leadership activities in primary prevention as other local organizations already have established network ties, and there is less *demand* for leadership activities as partner organizations fear that the medical focus of the institute will dominate the network that strives to support public health. The precise mix of need and demand may differ per country context, but the role of other leadership organizations and the feared domination of medical focus is likely to feature in other settings as well [42].

Secondly, while there is no demand for leadership activities from the UMC, there is a consensual demand for research activities. Paradoxically, on the one hand stakeholders ask for more evidence based knowledge, while at the same time they proclaim that this ‘universal knowledge’ does not fit the pragmatic concerns stakeholders face in practice. Community actors favor contextually embedded knowledge. This finding is likely to be relevant beyond this particular context in Utrecht. For example, Harris’ study (2021) of a US regional university identified a disconnect in research priorities between the university and the community [43].

Thirdly, the literature on collaborative governance points towards university hospitals as anchor *institutions*, while our study reveals that in practice images of the institution *as a whole–*‘the big medical hospital’ ‐ overshadow positive experiences with collaborative projects on primary prevention with individual professionals. Whilst individuals might be trusted for their ‘goodwill’ and affinity with primary prevention, on the institutional level the image of a detached medical ‘power player’ dominates. The internal dynamics of anchor institutions–how internal actors shape the overall (perception of) the institution ‐ is likely to feature by hospitals across the world, yet is an underexplored theme in the literature.

### Limitations of the research

This research is limited by several factors. First, our study concentrates on just one out of the eight regions. Because of the density of networks within this region, we identified our case as an extreme case example. This makes the case useful for identifying crucial mechanisms, but not necessarily representative for regions across the world. We should therefore be cautious with generalizing our findings to other contexts, although we expect that the mechanisms are likely to feature in other contexts as well.

Second, we identified quite a few incomplete surveys. While the combination of free name calling and the roster method increases measurement validity, it also extends the survey and the possibility that people drop out because of the time investment asked for to complete the survey. Giving the relatively high response rate and the opportunity to supplement the data with interviews and the workshop, we still feel some valid observations can be made.

### Implications

Following from the findings highlighted, our study has several implications. First, our study shows that UMCs are not necessarily viewed as natural allies in the fight for primary prevention. Many actors will primarily associate the UMC with patient treatment and academic knowledge. The top leadership have to actively promote the need for primary prevention, both internally and externally, and explain to their own staff and partners that collaboration is actually a key part of medical work [44–46]. Internally, this also implies that UMCs act more on primary prevention internally–for instance by standardly serving healthy food or incorporating physiotherapy within treatment trajectories.

Second, network activities should be *customized*. In some parts of the domain, on specific tasks, the UMC is granted more leeway and is actively looked to for initiative. Network partners are very clear in what they want the UMC to do (attracting attention, generating funding, developing new research methods), and what they do *not* want the UMC to do.

Finally, when the context of the regional network demands it, UMCs should consider positioning themselves as servants rather than leaders. In the case of the Utrecht, the focus could be on providing platforms for data collection, meeting points for experts, promotional support for prevention activities, and some work on helping to establish whether the collaboration has an impact. When other UMCs countries explore their particular local context they may also find that leading-by-serving gives them the best position for promoting public health.

### Suggestions future research

As future work, we suggest more comparative research, both between regions and countries. Our study focused on a single case of one of the prevention regions in the Netherlands. As each region has its own history and contextual specifics, future research should take into account the different ways in which collaboration on prevention has evolved, to trace how locality impacts granted activities and roles for UMCs. Further, national comparisons can be helpful in deepening our understanding of how collaborative efforts on primary prevention are initiated and coordinated in different OECD countries, and test if images of UMCs as powerful ‘medical institutions’ hold in other national contexts. As our study points to divergent perceptions of individual professionals and organizations, research could further explore the internal dynamics of anchor institutions.

## Supporting information

S1 File (DOCX)
