# Supplementary material for: What leadership role should University Medical Centers take in regional primary prevention networks? An interdisciplinary, multi-method analysis of a crowded stakeholder environment
Source: PLoS One. 2024 Jul 11;19(7):e0305262. doi: 10.1371/journal.pone.0305262 (PMC11238967; doi:10.1371/journal.pone.0305262)
Supplement: S1 File — (DOCX) [file pone.0305262.s001.docx]

# Survey^[[1]](#footnote-1)^

## Introduction

In the […] region, there is a strong focus on prevention, with numerous organizations actively promoting and working on a healthy region. For instance, the [name network] facilitates regional collaboration among policy, practice, educational, and knowledge institutions. Additionally, various other networks, both large and small, foster cooperative efforts. This questionnaire seeks to explore existing collaborative relationships in the region, with a focus on primary, universal prevention aimed at enhancing the overall population's health.

The questionnaire also specifically focuses on the role of the [umc]. As a healthcare, research, and training institution, the umc aims to contribute to primary prevention. [Further information about the research project and future participation] Thank you for your cooperation!

Question 1a. Which organization do you work for? [open answer]

Question 1b. Please tick the fields/roles that best describe your position. Multiple answers possible.

- *Policy advice*
- *Policy making*
- *Primary care (e.g. General Practitioner, Social work)*
- *Expert of experience*
- *Research (e.g., university, advisory council, research institution)*
- *Independent consulting (e.g., consulting firm, self-employed)*
- *Education*
- *Project management*
- *Social Domain (e.g., debt relief, social housing, employment consultant)*
- *Second-line care (e.g., hospital, clinic, mental health organization)*
- *Other, namely [answer]*

## Prevention activities of own organization

Question 2. In this study, the focus is on primary, universal prevention aimed at health promotion. What themes within this type of prevention does your organization focus on? Multiple answers possible.

- *Supporting lifestyle interventions (e.g. around nutrition, exercise, alcohol/drug use, smoking)*
- *Reaching a specific target group (e.g. young people, elderly, migrants, at-risk groups)*
- *Improving the living environment (e.g. healthy school, healthy neighbourhood, healthy urban living)*
- *Using E-health for prevention (use of information and communication technology)*
- *Using big data and/or artificial intelligence in prevention*
- *Developing research methods focused on prevention*
- *Collecting data on prevention (monitoring)*
- *Educating and training professionals in the field of prevention (e.g. in health care, education, sports, social work)*
- *Evaluating interventions focused on prevention*
- Other, namely [answer]

Question 3. To what extent are you satisfied with the results achieved in the various areas of prevention? [five-point Likert scale, per area of prevention]

| **Area of prevention** | **Answer options** |
| --- | --- |
| Supporting lifestyle interventions (e.g. around nutrition, exercise, alcohol use, smoking) | *[Very dissatisfied - dissatisfied - neutral - satisfied - very satisfied – not applicable]* |
| Reaching a specific target group for prevention (e.g. young people, elderly, migrants, at-risk groups) |  |
| Improving the living environment (e.g. healthy school, healthy neighborhood, healthy urban living) |  |
| Using E-health for prevention (use of information and communication technology) |  |
| Using big data and/or artificial intelligence in prevention |  |
| Developing research methods focused on prevention |  |
| Collecting data on prevention (monitoring) |  |
| Educating and training professionals in the field of prevention (e.g. in health care, education, sports, social work) |  |
| Optional: other, namely [answer] |  |

Question 4. What do you see as the biggest challenge in primary, universal prevention in the […] region? [open question]

## Collaboration with other organizations

Question 5. Name a maximum of three organizations in the […] region with which your organization collaborates most frequently and intensively on prevention. Describe the activities you undertake together. [up to three answers possible]

1. *Organization: [answer] + activity: [answer]*
2. *Organization: [answer] + activity: [answer]*
3. *Organization: [answer] + activity: [answer]*

Question 6. Name up to three organizations you do not yet collaborate with in the area of prevention, but would like to collaborate with. Describe the intended activities. [up to three answers possible]

1. *Organization: [answer] + intended activity: [answer]*
2. *Organization: [answer] + intended activity: [answer]*
3. *Organization: [answer] + intended activity: [answer]*

Question 7. How often do you have contact with the organizations listed below regarding universal primary prevention? [six-point Likert scale, per organisation one answer possible]

| **Organizations** | **Answers** |
| --- | --- |
| Central government | *[Never or almost never - about once a year - about once a month - about once a week- several times a week]* |
| Public health service |  |
| Municipality/municipalities |  |
| Hospital(s) |  |
| Second-line care institution(s) |  |
| First-line care institution(s) |  |
| Research institution(s) |  |
| Educational institution(s) |  |
| Sport association(s) |  |
| Welfare organization(s): neighbourhood team(s), community centre(s), clubhouse(s), etc. |  |
| Civic initiative(s) |  |
| Other: namely [answer] |  |

Question 8. For the various activities, please indicate the organizations with which you cooperate in these areas. [multiple answers possible]

| **Activities** | **Answers** |
| --- | --- |
| Supporting lifestyle interventions | *[Central government - public health service (GGD) – municipality - hospital(s) - second-line care institution(s) - First-line care institution(s) - research institution(s) - educational institution(s) - sport association(s) - welfare organization(s) - civic initiative(s) – not applicable]* |
| Achieving specific target group |  |
| Improving the living environment |  |
| Deploying E-health |  |
| Developing research methods focused on prevention |  |
| Developing big data / artificial intelligence in prevention |  |
| Collecting data on prevention (monitoring) |  |
| Training/educating people |  |
| Evaluating interventions aimed at prevention |  |
| Others, namely: *[answer]* |  |

Question 9. Is your organization part of one or more networks dealing with prevention in the […]region? [one answer possible]

- *Yes*
- *No*
- *I don’t know*

Question 10. Indicate which network or networks focused on prevention your organization is part of. [open question]

## Contribution umc

The following questions will focus specifically on the role of umc […] within networks focused on primary, universal prevention.

Question 11. With which division of the umc do you have contact/cooperate in the area of prevention? If there is no collaboration, click: 'no collaboration'. If you do not know which division the collaboration falls under, click: 'don't know'. [multiple possible]

- *Radiology and Oncology (Nuclear health care)*
- *Heart & Lungs*
- *Surgery*
- *Brain*
- *Internal Medicine and Dermatology*
- *[Research] Center for Health Sciences and Primary Care*
- *Children*
- *Laboratories, Pharmacy and Biomedical Genetics*
- *Vital Functions*
- *Woman & Baby*
- *Don't know*
- *No collaboration*

Question 12. If there is cooperation. What do you collaborate on with the umc? [open question]

Question 13. We now ask you to prioritize. Indicate in order of importance which theme would be most beneficial to your organization if the umc […] were to address it. Put at 1 the theme where umc […] can be of most value to you and at 8 the theme where umc […] can be of least value to you. [rating the answer options from 1 to 8]

- *Support lifestyle interventions (e.g. around nutrition, exercise, alcohol, smoking)*
- *Reach a specific target group for prevention (e.g. youth, elderly, migrants)*
- *Improving the living environment (e.g. healthy school, healthy neighbourhood)*
- *Using E-health for prevention (use of information and communication technology)*
- *Developing research methods focused on prevention)*
- *Collecting data on prevention (monitoring)*
- *Educating and training professionals in the field of prevention (e.g. in health care, education, sports, social work)*
- *Evaluating interventions aimed at prevention (evidence based intervention)*

Question 14. Rank which task would be valuable to your organization if umc […] were taking responsibility for it. Put at 1 the task you find most important and at 9 the task you find least important. [rating the answer options from 1 to 9]

- *Create awareness for issues*
- *Bringing parties together*
- *Bundle and sharing information*
- *Sharing resources*
- *Investigating innovative interventions and techniques*
- *Making policy or plans*
- *Offering services*
- *Monitoring and evaluating approach*
- *Setting up (consultation) structures*

Question 15. What would you advice umc […] when it comes to prevention in the coming years? [open question]

## Further Participation in Research

Question 16. Finally, we ask you a question regarding the continuation of this research. Are you willing to participate in an individual or group discussion to deepen the outcomes of this study? If you are willing to participate, please enter your e-mail address in the column below. Your information will be kept confidential. Only the researchers will have access to this information. [one answer possible]

- *Yes, I am willing to participate in an individual or group discussion. Enter mail address: [answer]*
- *No*

1. This survey has been translated. Original language: Dutch [↑](#footnote-ref-1)
